# Exploring the Multifaceted Impact of Acne on Quality of Life and Well-Being

**DOI:** 10.7759/cureus.52727

**Published:** 2024-01-22

**Authors:** Priyashman Nandy, Tripti Shrivastava

**Affiliations:** 1 Medicine, Jawaharlal Nehru Medical College, Datta Meghe Institute of Higher Education and Research, Wardha, IND; 2 Physiology, Jawaharlal Nehru Medical College, Datta Meghe Institute of Higher Education and Research, Wardha, IND

**Keywords:** hormones, face, skin, scars, sebum, acne vulgaris

## Abstract

A suitable portion of the total population still suffers through acne vulgaris, a widespread dermatological illness that mostly affects teens and young adults. Although acne is typically considered to be a cosmetic problem, recent research has concluded that certainly it has a significant role on impacting many physiological aspects of human health. This thorough investigation attempts to examine the intricate effects of acne on human physiology, taking into consideration both systemic and local effects. The study synthesizes research from a number of scientific disciplines, including dermatology, endocrinology, immunology, and psychoneuroimmunology. It investigates the complex interrelationships between several factors, such as sebum production, follicular hyperkeratinization, hormone imbalance, and *Propionibacterium acnes* colonisation, that lead to the development of acne. The study also sheds information on the intricate physiological mechanisms, oxidative stress, and immune response that contribute to the aetiology of acne. Along with it, the review investigates the relationship between endocrine problems and acne, emphasizing hormonal anomalies and their possible role in acne severity. The psychological effects of acne are also discussed, including the psychological problems, concerns with self-esteem, and decreased quality of life that acne sufferers encounter. On comprehensively examining the diverse physiological aspects affected by acne, this review provides a foundation for future research endeavours and informs the development of targeted therapeutic interventions. Ultimately, the integration of multidisciplinary approaches will enable healthcare professionals to address the physiological complexities of acne and improve the overall well-being of individuals affected by this common skin condition.

## Introduction and background

Lately, acne research has focused on understanding the local manifestations and mechanisms within the skin. However, recent studies have expanded the scope to investigate the broader impact of acne on human physiology. It has become increasingly evident that acne is not solely confined to the skin but affects various physiological systems, including the endocrine, immune, and psychological systems. Acne lesions typically result from the interaction of multiple elements, including the pilosebaceous follicles, sebum production, colonisation by *Cutibacterium acnes* (formerly known as *Propionibacterium acnes*), and the inflammatory response [1]. Moreover, Caucasians are found with mild acne, but generally, Asians and Africans have severe acne [2]. Multiple factors are responsible for acne vulgaris disease, and one of them includes skin microbes. The skin follicles housing the skin microbiome contain a wide array of microorganisms. Notably, *Propionibacterium acnes* and *Malassezia* spp. play a major role in the development of acne. Their influence on sebum production, comedone formation, and inflammatory responses is essential in the occurrence of acne [3].

The endocrine system is one of the key physiological aspects influenced by acne [4]. Hormonal imbalances, precisely an increase in androgens, have been associated with the development and sustenance of acne. Androgens govern the growth and operation of both the male and female reproductive systems, as is well known [5]. Testosterone, which is largely generated by Leydig cells in the male testis, is the major androgen in the blood. Although the male testis's Leydig cells may also release testosterone, these cells are the main source of circulating androgens. To understand the physiological mechanisms underpinning acne pathogenesis, it is essential to comprehend the complex hormonal shifts and their effects on sebum production, follicular hyperkeratinization, and inflammation [6].

Furthermore, metabolic disorders [7] and systemic inflammatory conditions have been linked to acne growth. Chronic low-grade inflammation, often observed in individuals with acne, can lead to the development of conditions such as metabolic syndrome, insulin resistance, and cardiovascular diseases. Talking about insulin resistance, high glycemic load diets encourage the development of acne vulgaris via elevating insulin-like growth factor 1 (IGF-1) levels. Lesions of acne are less common in those who have acne and follow diets with a low glycemic load compared to those with a high glycemic load. This is due to the lower amount of carbs in low glycemic load diets [8]. Exploring the intricate interplay between inflammation, oxidative stress, and systemic physiology is essential for uncovering the potential long-term consequences of acne on overall health.

In the case of acne, psychological ramifications are equally important. Acne may significantly impact someone's quality of life generally, body image, and self-esteem [9]. Acne sufferers frequently feel psychological problems, which can have significant effects on social interaction, interpersonal relationships, and mental health. Understanding acne's psychological impact is crucial to offering complete treatment and support for persons suffering from this illness. Research already conducted reveals that acne has a variety of impacts, including a poor correlation with health-related quality of life [10].

According to epidemiological research, acne often develops throughout adolescence and may last until the early 30s. Acne is seen to afflict men more frequently than women, and metropolitan populations tend to have greater prevalence of the condition than rural ones. Furthermore, 20% of people with acne vulgaris have severe acne scars, which have a substantial negative influence on their quality of life owing to the physical harm and altered look. Additionally, certain racial groups can be more susceptible to acne than others [11]. Hyperpigmentation is typically more likely to occur in those with darker skin tones. Acne can sometimes occur in newborns, although it usually goes away independently after a while [12]. The colonisation of the bacteria *Propionibacterium* from hair follicles on the chest, neck, and back induces the growth of acne [13]. Effectively, early colonisation of *Propionibacterium acnes* and the family history might have important roles in acne vulgaris. However, it is still not clear exactly what is the cause of this disease [14]. An unproven factor for acne vulgaris could be diet. Recently, high-carbohydrate food and dairy products have been found to be aggravating factors for acne vulgaris. Acne scars are severe; one can even last the whole life, and that leads to depression, social isolation, and suicidal ideation [15].

Given the diverse physiological implications of acne, a multidisciplinary approach is necessary to explore its impact on human physiology comprehensively. Integrating findings from dermatology, endocrinology, immunology, genetics, and psychoneuroimmunology enables a holistic understanding of the complex interactions between acne and various physiological systems.

## Review

Search methodology

**Figure 1:**
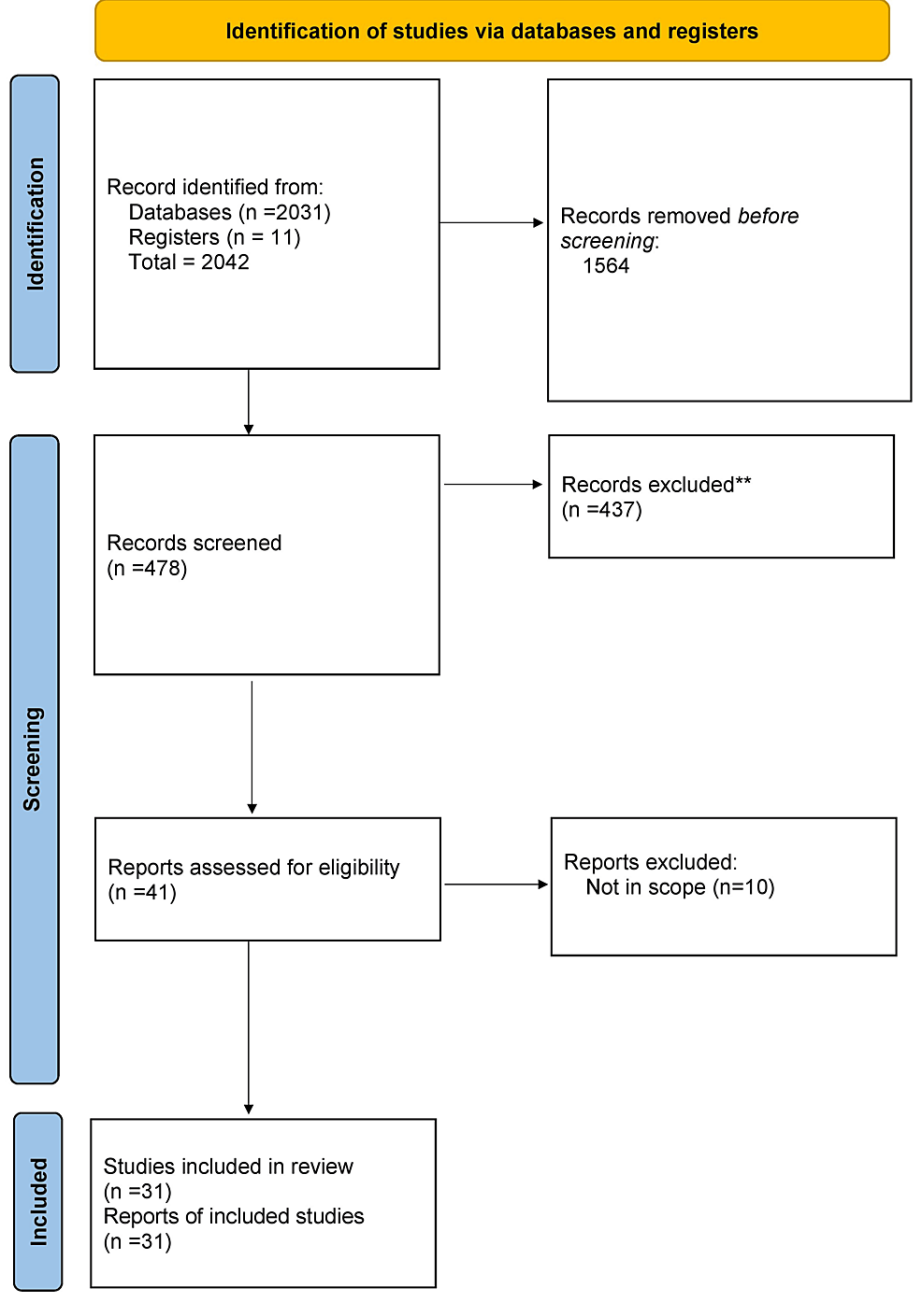
PRISMA flowchart for literature search PRISMA: Preferred Reporting Items for Systematic Reviews and Meta-Analyses

We reviewed PubMed, Google Scholar, and Web of Science databases for articles on Exploring the Multifaceted Impact of Acne on Quality of Life and Well-Being. We included as many relevant studies as possible using the medical subject heading phrases "impact of acne on life" and "effects of acne vulgaris" as well as various keyword combos. To identify potential additional records from other sources, literature search was conducted to locate case-control studies and meta-analyses. Figure 1 shows the Preferred Reporting Items for Systematic Reviews and Meta-Analyses (PRISMA) flowchart for literature search.

Research on the impact of acne on human physiology utilizes various methodologies to investigate the multifaceted aspects of this condition. Here are some commonly employed research methodologies and approaches in this field.

Observational Studies

Numerous research have examined how different foods' glycemic loads and glycemic indices affect acne sufferers, and they have found that people who consume low glycemic load diets often experience fewer acne lesions than those who consume high glycemic load diets. The involvement of dairy, especially the whey proteins contained in milk, as possible contributors to acne formation has been highlighted in research on the connection between diet and acne, outweighing the impact of real fat or dairy intake levels. Additionally, studies looking at the effects of the fatty acids omega-3 and linoleic acid consumption in those with acne have shown diets that include fish and healthy oils like olive oil might be good for those with acne difficulties [16].

Clinical Trials

Randomized controlled trials (RCTs) are carried out to assess the effectiveness and safety of various acne therapies and their effects on physiological parameters. Numerous treatments, like microneedling monotherapy used for removing or fading the scars and topical or systemic drugs, are frequently used in these studies, and they often track changes in sebum production, inflammatory levels, or hormonal balance [17].

Histopathological Analysis

In order to understand the structural and cellular changes brought on by acne, skin samples from people with lesions are scrutinised under a microscope. Sebaceous gland abnormalities, anomalies in hair follicles, and inflammatory skin conditions can all be found by histopathological investigation [18].

Hormonal Analysis

The hormonal abnormalities linked to acne require a thorough grasp of endocrine research. The levels of androgens (such as testosterone), IGF-1, sex hormone-binding globulin (SHBG), and other important hormones in acne patients are measured and analysed using hormonal assays [19].

Genetic Studies

Genetic studies seek to pinpoint genetic variations and polymorphisms linked to acne severity and susceptibility. Candidate gene studies and genome-wide association studies (GWAS) examine the genetic makeup of people with acne to find potential genetic markers [20].

Psychological Studies

Psychological and quality-of-life assessments are carried out to determine how acne affects social interaction and mental health. Questionnaires, scales, and interviews are used to measure psychosocial factors, including self-esteem, body image, sadness, and anxiety [21].

Combining these study techniques enables a thorough comprehension of how acne affects human physiology. The complicated relationships between acne and physiological systems can be better understood by integrating clinical, histological, immunological, genetic, and psychological studies. Acne has always been seen as primarily a cosmetic issue, but new study has shown that it affects many other elements of human physiology in addition to its outwardly obvious symptoms. It is crucial to comprehend the complex interaction between acne and human physiology to create efficient management plans and enhance the general well-being of people with this illness.

Classification

Fungal acne (pityrosporum folliculitis) is characterised by the accumulation of yeasts in hair follicles. These could be swollen and irritating [22]. The other type could be cystic acne which is observed as deep nodules and pimples packed with pus. They could leave scars [23]. Adults with high sebum production lead to hormonal acne which further leads to clogging of pores [24]. Nodular acne is a severe kind of acne that leaves lumps under the skin that are sensitive and nodular in appearance [25].

Symptoms

Pimples, known as pustules, are raised bumps containing pus. Papules are small, discoloured spots, often red or darker than the surrounding skin tone. Clogged pores with a dark or black surface characterise blackheads. Whiteheads are formed when pores are clogged with a white or flesh-coloured surface. Nodules are substantial and painful lumps that develop beneath the skin. Cysts are fluid-filled painful lumps beneath the skin's surface.

Effects of acne vulgaris

Physical Effects

Pimples, blackheads, whiteheads, and occasionally cysts are the main physical effects of acne, which predominantly affects the skin. Though painful and irritating, it often does not represent a serious risk to physical health.

Psychological Effects

Acne may significantly affect people, especially throughout adolescence, when social growth depends on self-esteem and body image. Due to worries about their appearance, many people who have acne may have decreased self-confidence, increased social anxiety, and even melancholy.

Scarring

Severe acne occasionally results in scarring, which might have long-term aesthetic repercussions. Scarring can cause mental anguish even when it has no direct effect on physical health.

Secondary Infections

Acne lesions that are left untreated or improperly managed occasionally become infected, which can result in more severe problems. However, these occurrences are uncommon and are frequently avoidable with appropriate acne treatment and cleanliness.

Effect on Quality of Life

Acne can reduce a person's quality of life by making them socially withdrawn, avoid social situations, and participate less in once-valued hobbies.

Degree of acne vulgaris

Acne predominantly affects the centrofacial areas, back, upper trunk, and deltoid region, giving rise to various types of skin lesions, starting with comedones, and there are two types of comedones: open and closed. Open comedones occur when the pilosebaceous orifice on the skin surface becomes blocked with sebum, whereas closed comedones form when a combination of keratin and sebum obstructs the pilosebaceous orifice below the skin surface. The grades of acne with their severity are listed in Table 1.

**Table 1:** Degree of acne vulgaris

Grades	Severity
1	Formation of comedones
2	Small papules with redness are the hallmark of inflammatory lesions (erythema)
3	Pustules
4	Numerous pustules can merge, leading to the formation of nodules and cysts

Comedones, papules, pustules, and rarely nodules or cysts grow on the skin due to acne. Sebaceous glands, hair follicles, hormonal variables, microbial colonisation, and immunological responses all play a role in the multifactorial aetiology of acne. Inflammation, follicular hyperkeratinization, and the expansion of *Propionibacterium acnes* within the pilosebaceous unit are all influenced by these interconnected processes.

Treatment/management of acne

On using the method of topical therapy, benzoyl peroxide or other topical antibiotics are frequently used with topical retinoids such as retinoic acid, adapalene, and tretinoin in monotherapy. The most efficient comedolytic drug is retinoic acid, offered in cream and gel formulations with strengths of 0.025%, 0.05%, and 0.1% [26]. Also, adapalene and topical benzoyl peroxide are currently available together, acting as both a comedolytic and antibacterial preparation. In gel form, this mixture is offered in concentrations of 2.5%, 4%, and 5% [27]. Additionally, antimicrobial and comedolytic azelaic acid is available as 15% or 20% gel. It can also be used for acne pigmentation caused by inflammation, and topical dapsone can treat comedonal and papular acne. Still, in the case of G6PD-deficient people, it raises some queries.

The systemic therapy for treatment will include doxycycline as an antibiotic and anti-inflammatory, typically at a dosage of 100 mg twice daily. By controlling the release of free fatty acids, it prevents inflammation. Also, a daily dose of minocycline (50 mg and 100 mg), administered in pill form, will help once a day. Isotretinoin is also one of the most advised medications. Isotretinoin controls inflammation, pilosebaceous epidermal hyperproliferation, and sebum production by affecting *P. acnes*.

However, it might result in negative consequences including cheilitis, dryness, and hair loss. Especially for men, spironolactone (25 mg/day) is very helpful. It functions by decreasing the production of androgens and preventing testosterone from doing its job. It is crucial to avoid pregnancy if given to females, though, as it might feminise the baby [28]. Furthermore, chemical peels, trichloroacetic acid, derma rollers, microneedling, and fractional CO2 lasers are a few of the numerous scar treatments available [29,30]. Oral contraceptives primarily function to prevent pregnancy in non-invasive methods, but they have been found useful in controlling acne in premenopausal women as they suppress the levels of luteinizing hormone (LH) and follicle-stimulating hormone (FSH), which are the endocrine factors for the development of acne [31]. Preventive measures for acne vulgaris have been listed below in Table 2.

**Table 2:** Preventive measures to avoid acne vulgaris

Sr. no.	Preventive measures for acne vulgaris
1	Perform daily cleansing of your face using a gentle facial cleanser and warm water.
2	Apply an oil-free moisturizer regularly.
3	Choose makeup products labelled "noncomedogenic" and make it a habit to remove makeup before bed.
4	Refrain from touching your face with your hands.

Generally, most patients have favourable outcomes following acne treatment. However, for many individuals, acne can result in persistent scars. Educating patients about the importance of not manipulating the lesions and seeking timely care can aid in preventing these scars. Unfortunately, once acne scars have developed, the available treatments may not yield the most optimal results. The summary of articles included in this review is listed in Table 3.

**Table 3:** Summary of articles included in this review IGF-1: insulin-like growth factor 1; RCM: reflectance confocal microscopy; OCT: optical coherence tomography

Author	Year	Findings
Oge' et al. [1]	2019	*Cutibacterium acnes*, a type of acne, is a common chronic skin disease affecting 50 million people annually. Treatment options include topical retinoids, systemic antibiotics, and isotretinoin for mild to severe acne, with further research needed for physical and complementary therapies.
Leung et al. [2]	2021	Acne vulgaris, a condition affecting 9.4% of the global population, is most common in adolescents and young adults, with male predominance and female prevalence in adulthood along with the fact that Caucasians are more likely to have mild acne, while Asians and Africans are more likely to have severe acne.
Xu and Li [3]	2019	Acne vulgaris is a chronic skin disorder influenced by various factors, including skin microbes like *Propionibacterium acnes* and *Malassezia* spp. These microorganisms influence sebum secretion, comedone formation, and inflammatory response. Antibiotics targeting *P. acnes* have been widely prescribed for acne treatment. Understanding the skin microbiome and its effects on antibiotic treatment is crucial for clinicians.
Nguyen and Tollefson [4]	2017	Endocrine disorders, particularly acne vulgaris, significantly impact adolescents' quality of life, prompting clinicians to explore hormonal therapies as a potential alternative to antibiotics due to concerns about antibiotic stewardship.
Kircik [5]	2021	Androgens, a key factor in acne pathogenesis, are stimulated by sebaceous gland growth and sebum production. Clascoterone, a topical androgen antagonist, addresses these issues by binding to androgen receptors in sebaceous glands, reducing acne lesions without systemic effects.
Rezaković et al. [6]	2012	Follicular hyperkeratinization, inflammation, and androgen stimulation are key factors in acne, suggesting a potential link between diet and acne. Studies show low incidence in non-Western societies, suggesting diet may mediate inflammation, oxidative stress, and hormonal imbalance, potentially promoting preventive or therapeutic effects.
Franik et al. [7]	2018	Metabolic disorders can significantly impact acne vulgaris in women with polycystic ovarian syndrome, necessitating further investigation into the impact of these conditions.
Andreadi et al. [8]	2022	Consuming diets with a low glycemic load can reduce acne lesions, as they contain fewer carbohydrates. Studies show that individuals with acne who consume diets with a high glycemic load have fewer acne lesions, as a low glycemic regimen can significantly reduce serum IGF-1 concentrations.
Davern and O'Donnell [9]	2018	Social factors, such as stigma, play a significant role in the unpredictable and challenging symptoms experienced by acne sufferers, highlighting the need for comprehensive understanding and management strategies.
Lasek and Chren [10]	1998	Skindex measures skin disease's impact on patients' quality of life, with higher scores indicating greater effects, based on clinical severity and patient responses.
Sutaria et al. [11]	2023	Adolescent acne affects males more, while postadolescent acne primarily affects females. Urban populations are more affected, with Asians, Africans, and darker skin groups experiencing varying severity.
Özçelik et al. [12]	2018	Acne vulgaris, a common skin disease, is most prevalent between 14 and 17 years old, with 40% in girls. Early-onset puberty may cause it earlier, with a 36.5% prevalence rate in a study involving 8298 subjects.
Williams et al. [13]	2012	*Propionibacterium acnes*, a bacterium responsible for acne, causes inflammation and bacterial colonisation of hair follicles. Early colonisation and family history contribute to the disease, but treatment remains unclear. Acne can persist into adulthood, affecting self-esteem and posing challenges for treatment.
Schnopp and Mempel [14]	2011	Acne vulgaris is a common inflammatory skin disease affecting all age groups, with prepubertal acne being rare. It can be mild or severe, with early intervention needed. Etiopathogenesis is unknown, with family history and lifestyle factors being key risk factors. Treatment includes topical monotherapy, combination treatments, and systemic therapy.
Cooper and Harris [15]	2017	Severe acne, a chronic inflammatory disease, can cause long-lasting psychosocial effects, including depression, social isolation, and suicidal ideation. Despite various treatments, oral isotretinoin remains the most effective. Early, effective treatment with systemic therapy is recommended for moderate to severe cases.
Baldwin and Tan [16]	2021	Acid consumption in diets has been linked to acne pathogenesis, with low glycemic load diets reducing acne lesions. Studies show omega-3 and omega-6 fatty acid intake from fish and healthy oils benefits acne patients. Further research is needed to understand probiotic effects on acne treatment.
Shen et al. [17]	2022	Acne scarring, a cosmetic issue and psychological health risk, is often treated with microneedling, a treatment that has been compared in randomized controlled trials for efficacy and safety.
Fuchs et al. [18]	2019	The unique follicular infundibulum characteristics and degree of inflammation that were linked to the severity of acne were revealed by the combined use of RCM and OCT. In order to assess the effectiveness of treatment and support clinical acne grading, imaging techniques may be used in future trials.
Shaw [19]	2002	Hormonal therapies play a crucial role in treating acne, focusing on inhibiting androgen expression. These therapies, including combination oral contraceptives and androgen receptor blockers, are used to reduce circulating androgens from ovarian and adrenal sources. However, the effectiveness of these therapies in treating androgenetic alopecia in men remains largely unknown.
Navarini et al. [20]	2014	The genetic basis of acne susceptibility has been extensively studied, with heritability estimates reaching 81%. Genome-wide association studies have provided valuable insights into this genetic determinant.
Stamu-O'Brien et al. [21]	2021	Acne has a substantial psychological impact and, although common and nonthreatening, requires comprehensive treatment to enhance the patient's skin and self-esteem.
Rubenstein and Malerich [22]	2014	*Malassezia* folliculitis, a fungal acneiform condition, often misdiagnosed as acne vulgaris, can persist without resolution with typical acne medications, requiring oral antifungals and combination treatments.
Marynick et al. [23]	1983	Experimental studies of men and women with cystic acne resistant to conventional therapy revealed androgen excess. Treatment with dexamethasone or Demulen (G.D. Searle, Skokie, Illinois, United States) resulted in resolution or improvement in 97% of women and 81% of men.
Bagatin et al. [24]	2019	Adult female acne, affecting women over 25, is a chronic condition influenced by genetic and hormonal factors, causing psychological and social impacts.
Khammari et al. [25]	2019	Inflammatory nodules and scarring are the hallmarks of severe nodular acne. There has not been enough research done on their natural evolution and duration.
See et al. [26]	2018	Topical retinoids play a crucial role in managing acne vulgaris in Asian patients, despite their underutilization due to sensitivity and lack of experience. Optimizing use can improve adherence and patient satisfaction.
Kosmadaki and Katsambas [27]	2017	Azelaic acid, a potent antioxidant, is a key component in the treatment of acne, enhancing its efficacy and safety. Its use in combination with other treatments like benzoyl peroxide prevents bacteria resistance, while retinoids address both comedonal and inflammatory acne lesions, offering a more comprehensive approach.
Isvy-Joubert et al. [28]	2017	The study evaluated the efficacy of spironolactone in women with acne, focusing on its role in regulating sebaceous gland activity. Results showed that low-dose spironolactone is a valuable alternative, especially in cases where oral isotretinoin has failed. The study also suggests using third- or fourth-generation oral contraceptives.
Yadav and Gupta [29]	2018	Subcision is a common procedure for depressed acne rolling scars, causing fibrotic strands to break up, leading to neocollagenesis, hematoma formation, and tissue trauma.
Connolly et al. [30]	2017	The treatment of acne vulgaris involves an algorithmic approach categorized into erythematous and atrophic scars, addressing the cellular sequelae and ongoing clinical trials.
Evans and Sutton [31]	2015	Oral contraception is a popular, non-invasive method for pregnancy prevention, the control of acne, hirsutism, dysmenorrhea, and irregular menstruation, with generic formulations offering lower cost and efficacy.

## Conclusions

This study's objective is to evaluate acne's complex effects on human physiology objectively. This study aims to illuminate the complex links between acne and physiological processes by integrating current literature and using various research approaches. The results of this study will further enhance our knowledge of acne as a systemic disorder, make it easier to create specialised therapy approaches, and eventually enhance the general well-being of acne sufferers. At the time of my most recent update in September 2023, acne was widely regarded as a skin ailment that often affects many people, particularly throughout adolescence and the early stages of adulthood. Acne can significantly affect a person's physical appearance and self-esteem, but it seldom has a serious or life-threatening effect on their general health. It's crucial to keep in mind that everyone's experiences with acne are unique and that it can occasionally cause emotional anguish and mental health issues like anxiety and despair.
